# How can artificial neural networks approximate the brain?

**DOI:** 10.3389/fpsyg.2022.970214

**Published:** 2023-01-09

**Authors:** Feng Shao, Zheng Shen

**Affiliations:** Beijing Key Laboratory of Behavior and Mental Health, School of Psychological and Cognitive Sciences, Peking University, Beijing, China

**Keywords:** dual neural node, neuron type, energy source mode, hierarchical architecture, spike-time encoding, emergent computation, executive control to socially cognitive behavior

## Abstract

The article reviews the history development of artificial neural networks (ANNs), then compares the differences between ANNs and brain networks in their constituent unit, network architecture, and dynamic principle. The authors offer five points of suggestion for ANNs development and ten questions to be investigated further for the interdisciplinary field of brain simulation. Even though brain is a super-complex system with 10^11^ neurons, its intelligence does depend rather on the neuronal type and their energy supply mode than the number of neurons. It might be possible for ANN development to follow a new direction that is a combination of multiple modules with different architecture principle and multiple computation, rather than very large scale of neural networks with much more uniformed units and hidden layers.

## Introduction

Studies in ANNs have been the focus of contemporary society since the Image Net competition in visual object recognition was won by a deep neural network(Kietzmann et al., 2019; Kriegeskorte and Golan, 2019). Engineers dream of pursuing a class of brain-inspired machines. The situation of robots superseding humans in many functions may soon be a reality. Meanwhile, neurobiologists and psychologists wonder about progress in neural networks due to the great differences between ANNs and biological brains(Crick, 1989; Carlson et al., 2018).

This article reviews the contemporary theories and technical advances in three related fields: ANNs, neuroscience and psychology. ANNs were born mainly from more than two thousand years of mathematical theories and algorithms; over the past two hundred years, neuroscience has revealed more truths about the mysteries of the brain; and psychology has just passed its 143th anniversary of accumulating conceptions about conscious and unconscious cognitive processes. Cognitive sciences, encapsulating the disciplines of both natural and artificial intelligence, were founded in the mid-1970s to inquire into the mystery of intelligent substitutes, including humans, animals and machines. Although progress in these related fields is promising, a completed brain-inspired intelligent machine is far from the human brain. How can we reduce the distance between engineers’ dreams and reality? Some suggestions for brain simulation will be offered in the paper.

## Three generations of ANNs

Artificial neural networks simulate brain intelligence by mathematical equations, software or electronic circuits. As the first constituent sub-discipline of cognitive sciences, ANNs already have a 79-year history since the conception of the “neural unit” and “Hebbian synapse” as well as the first model of the perceptron neural network in the 1940s–1950s (Mcculloch and Pitts, 1943; Hebb, 1949; Rosenblatt, 1958). We review the developmental course of the discipline and divide its history into three stages that represent the view of the human brain and intelligence from the perspective of mathematics. The first generation of ANNs is linear logic networks; the second generation is connectionist networks, including parallel distributed processing (PDP) and deep neural networks (DNNs); and the third generation is spike neural networks (SNNs).

### The first generation of ANNs: Linear logic networks

The first sentence in the first ANN paper was “Because of the ‘all-or-none’ character nervous activity, neural events and the relations among them can be treated by means of propositional logic.”(Mcculloch and Pitts, 1943). Then, the authors described ten theorems and emphasized the calculus principle and academic significance in the following sentence in the last part of the paper: “Specification for any one time of afferent stimulation and of the activity of all constituent neurons, each an ‘all-or-none’ affair, determines the state.” It is worth paying attention to the keywords in the cited sentence, all-or-none and determine, which mean that perception is associated with the statistic separation of two-state affairs as a deterministic system. The second classical ANN paper stated, “When an axon of cell A is near enough to excite a cell B and repeatedly or persistently takes part in firing it, some growth process or metabolic change takes place in one or both cells such that A’s efficiency, as one of the cells firing B, is increased” (Hebb, 1949).

The first ANN perceptron model was a linear network model that comprised A units and R units. Eleven equations were used to analyze the model’s predominant phase through six parameters clearly defining physical variables that were independent of the behavioral and perceptual phenomena. As a result, the model was able to predict learning curves from neurological variables and likewise to predict neurological variables from learning curves. The author assertively wrote, “By the study of systems such as the perceptron, it is hoped that those fundamental laws of organization which are common to all information handling systems, machines and men included, may eventually be understood” (Rosenblatt, 1958). It is obvious for the classical writer to have too optimistic foresee for his linear network model. In fact, the linear model could not solve mathematic puzzles such as XOR partition. Therefore, a monograph (Minsky and Papert, 1969) claimed assertively that the investigation of linear networks must be only a game on paper. As a result, much foundation grant money was withdrawn, no longer supporting the projects.

### The second generation of ANNs: Connectionist networks

2-G ANNs began to be developed in the mid-1980s and can be divided into two periods: parallel distributed processing (PDP) and deep learning. In addition to the demand for ANN development, the demand for debugging in artificial intelligence (AI) also promoted the renaissance of ANNs in the 1980s.

A few dedicated scientists persisted in their neural network programs after 1969, their achievements provided a prelude for an ANN renaissance. For example, two neural network models, the discrete two-state model(Hopfield, 1982) and the continuous-response model (Hopfield, 1984), were published. A two-volume book (Rumelhart and McClelland, 1986) about PDP written by 16 experts as coauthors was spread quickly over the world. Many neural models and learning algorithms, such as the massively parallel network with hidden unit-Boltzmann machine (Ackley et al., 1985), error backpropagation (Rumelhart et al., 1986), competitive learning, neural Darwinism, adaptive systems, and self-organized learning, were created quickly during the 1980s–1990s. Meanwhile, connectionist modern supercomputers were developed, and very large-scale analog integrated circuits, analog neural computers, and applied electronic chips, such as electronic retina chips and see-hear chips, emerged.

It is worth discussing backpropagation algorithm (BP) due to its broad applications in the field of ANNs. Rumelhart et al. (1986) described the learning procedure and abstracted a universal δ-learning rule. The results in implementation of the learning algorithm demonstrate that there is error gradient descent in weight-space, so that network’s output reaches at its targeted value in a little by a little, meanwhile the error derivatives (*δ*) between the output and the desired result propagates back along the gradient descent. In fact, the efficiency of learning procedure usually is very low. For example, it is necessary more than several hundred even thousand turns of training for a very simple network with a hidden unit to solve a task of XOR function partition. BP is not a nonlinear model, error back-propagation is just an implicit nonlinear mapping without a really feedback loop. The number of hidden nodes has to be make sure by experience or testing without theoretical guideline. The authors wrote in the end of their paper that “the learning procedure, in its current form, is not a plausible model of learning in brains. However, …it is worth looking for more biologically plausible ways of doing gradient descent in neural networks.”

The studies in DNNs were initiated by the heuristic ideas: parallel distributed processing (PDP), and stochastic computing, for example, Boltzmann machine. Although its initial step began early in 1980s, it did not shoot a flash in scientific society until Alex-Net won image competition in 2012. Alex-Net was composed of 650,000 artificial neural units, consisted five convolutional layers, some of which were followed by max-pooling layers and three fully connected layers with a final 1,000 ways to output the results. It successfully recognized and classified 1.2 million high-resolution images by 60 million parameters in the ILSVRC-2012 competition (Krizhevsky et al., 2012). Recently, a deep CNN based Kaggle network won the contest to recognize real-time facial expression in the 5th ICEEICT (Sultana et al., 2021).

Beside the multiple hidden layers of network, a number of new algorithm has given DNNs a strong support, for example, convolutional algorithm, wavelet, and principal component analysis (PCA), increased high accuracy of feature extracting and pattern classification. The roles of traditional shallow models in pattern recognition had been changed in recent years, because of deep CNNs with strong learning ability—the deep learning-based object detection. The situation is based on some premises, such as the database of large scale networks had been accumulated during Image Net twice competitions in LSVRC-2010 and LSVRC-2012 (Deng et al., 2009); CNNs for speech recognition had been reported (Hinton et al., 2012); a design of regions with CNN features was successively applied (Girshick et al., 2014). In short, CNNs can learn object representations without the need to design features manually and can straightforwardly process visual or auditory complex objects without object dividing or component extracting. The deep learning-based object detection models and algorithms, covering different application domains, had been reviewed in detail (LeCun et al., 2015; Zhao et al., 2019; Naveen and Sparvathi, 2022). It is possible for the progress in studies of machine deep learning to promote ANNs serving social life and economic development. But there are many mysteries opening with regard to how does the CNNs learn the ability. The answer is that BP learning procedure trains CNNs approximating the desired output by the mechanism of error-backpropagation (Lillicrap et al., 2020). But the article did not make sure that the same mechanism exists also in the biological brain. They cited a number of references which belong to the studies in either neuroanatomy or neurophysiology, missing the dada in studies of neurobiology or oscillatory encoding. For example, the theory of oscillatory encoding claims that information communication between brain areas might be implemented by the dynamic synchronization of different rhythmic activities among neural networks. GABA inter-neurons by their inhibition lock up pyramidal neurons coupling with a network oscillation, then Neurogliaform cells (NGFCs) dynamically decouple neuronal synchrony between brain areas (Sakalar et al., 2022). It is interesting that action potentials of NGFCs decoupled pyramidal cell activity from cortical gamma oscillations but did not reduce their firing nor affect local oscillations. Thus, NGFCs regulate information transfer by temporarily disengaging the synchrony without decreasing the activity of communicating networks. Such attribute of NGFCs in biological brain seems to be alike of backpropagation in ANNs or CNNs, it regulates error gradient in weight-space by implicit feedback fashion, but does not disturb feedforward information transfer of ANNs. The result in comparison of the functional fashion between cortical NGFCs and backpropagation in ANNs supports the idea that mechanism of implicit backpropagation exists in both biological brain and ANNs.

**Figure 1:**
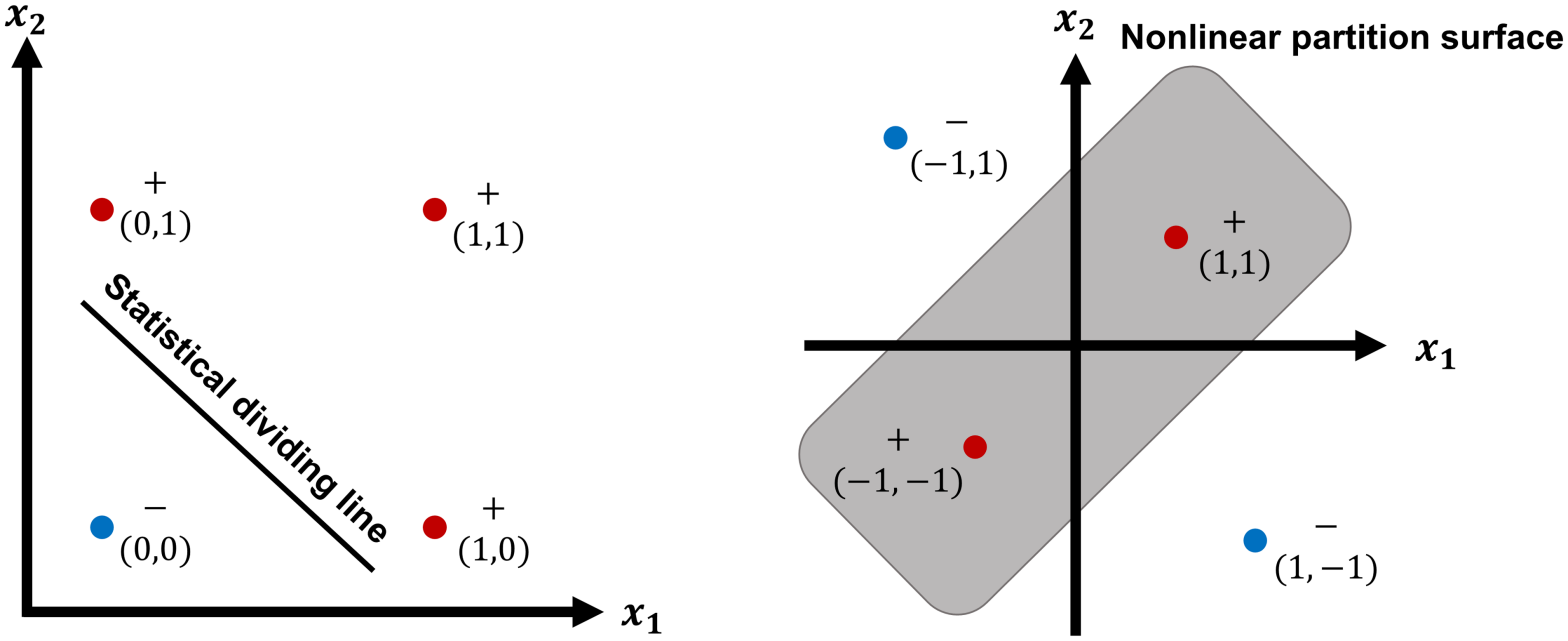
The linear dividing of OR function (left) and nonlinear partition of XOR function (right). XOR function is a propositional logic corresponding to OR function. As showing in the left plot, the results of OR function can be divided into two parts by a statistical dividing line, because the result is false (−) at the left side (blue point) of the dividing line, so long as two variable × 1 and × 2 are false (0,0); the results under the other 3 conditioning (0,1; 1,0; 1,1) are true (+, red points) at the up side of the line. The right plot shows nonlinear partition surface of XOR function. The results of XOR function are inside a surface (red points) by a closed curve, as the conditioning variable is, respectively, true (+,+) or false (−,–); the other two results (blue points) are outside of the partition surface, during the conditioning variable is, respectively, as −1, 1 or 1, −1.

Despite the great progress in ANNs, DNNs, and CNNs, as well as their rapid spread throughout the world, their weaknesses sometimes spoiled their reputation by spontaneously generating adverse effects, leading to some strange results. Fortunately, this phenomenon has already been solved. In addition, the many neural units and large parameters required by a deep neural network created a somewhat complicated problem because they not only wasted energy but also prevented the application of DNNs to pragmatic problems. Spiking neural networks have an advantage in decreasing both the unit numbers and energy needed (Figure 1).

### The third generation of ANNs: Spiking neural networks

1,2-G networks are composed of a deterministic system in which information transmission is determined by presynaptic and postsynaptic factors. Neurobiological achievements in three research fields, studies on the ion channel conductance of the neuronal membrane (Williams, 2004), studies on the probability of transmitter release from the presynaptic membrane (Tsodyks and Markram, 1997; Branco and Staras, 2009; Borst, 2010), and studies on spike timing-dependent plasticity (STDP; Song et al., 2000; Nimchinsky et al., 2002; Zucker and Regehr, 2002; Caporale and Dan, 2008; Losonczy et al., 2008; Spruston, 2008; Jia et al., 2010; Mark et al., 2017), produced a new conception of synaptic transmission and have attracted many experts to explore temporal coincidence (Montague and Sejnowski, 1994; Sejnowski, 1995; Markram et al., 1997; Roelfsema et al., 1997), noise sources (Maass, 1997; Roberts, 1999; Hopfield and Brody, 2004; Buesing and Maass, 2010; McDonnell and Ward, 2011), spiking neural networks (SNNs) and stochastic computing (Silver, 2010; Hamilton et al., 2014; Maass, 2014; Maass, 2015; Tavanaei and Maida, 2017) since the 1990s.

**Figure 2:**
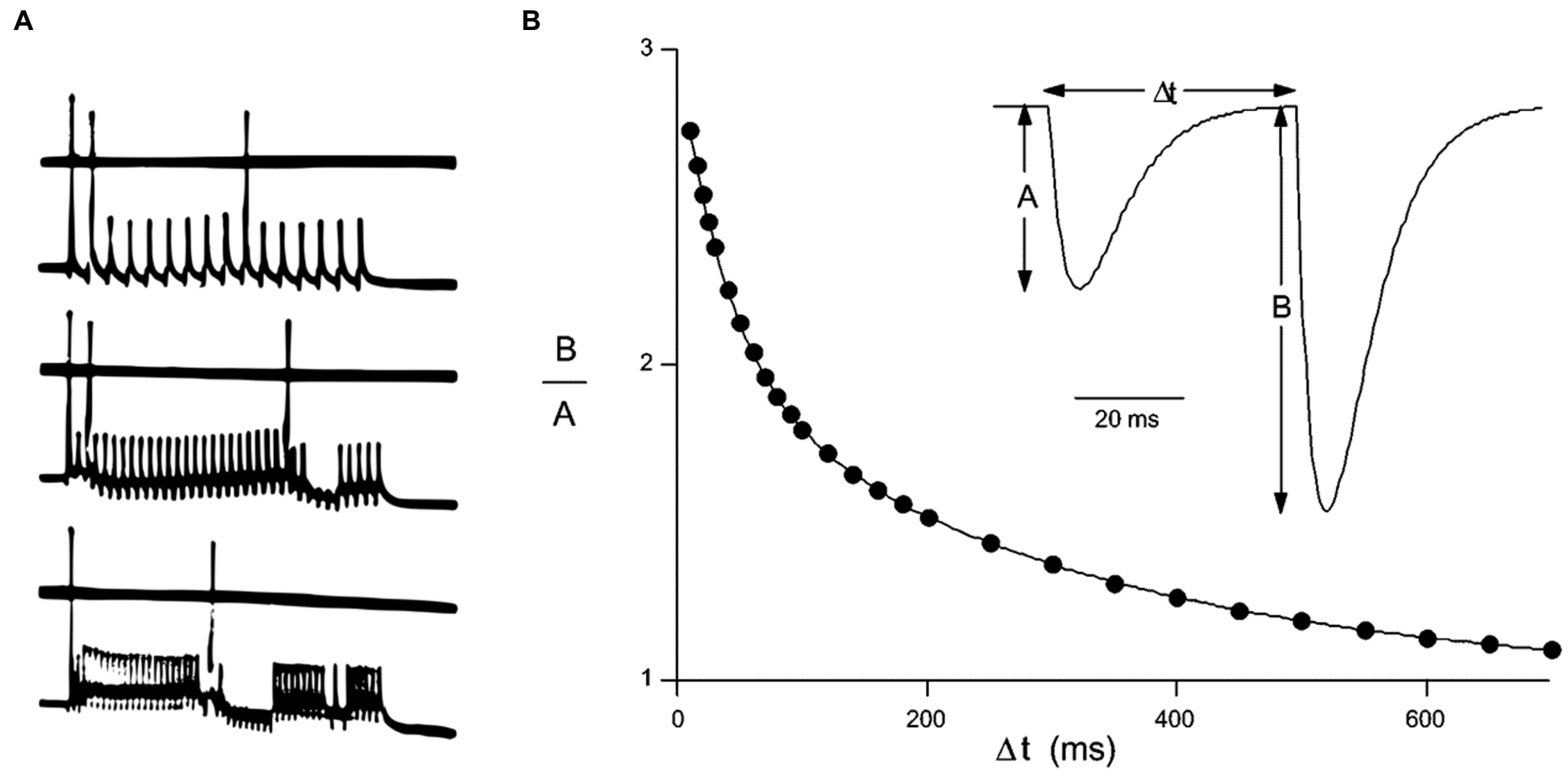
Three categories of neuronal encoding **A**. Rate encoding by the firing frequency of a neuron, demonstrating approximately 150, 300, and 450 Hz induced by different stimulus strength in plot a (modified from Eccles, 1953) **B**. Paired-Pulse Ratio encoding (PPR) and spike-time encoding are implemented, respectively, by the ratio of amplitude *B* to *A* or Δ*t* of spike-timing in plot b [modified from Zucker and Regehr, 2002 by permission].

Such as showing in Figure 2, there are three categories of neuronal encoding: rate encoding, paired pulses ratio (PPR) encoding and spike-time encoding. Rate encoding has been adopted to present neuronal exciting level since 1930s, PPR encoding and spike-time encoding are discussed in the field of neurophysiology during 1990s. PPR encoding has been usually used to classify the neurons, Spike-time encoding employs the lengths of inter-spike intervals (Δ*t*) to encode and transmit information (Sejnowski, 1995; Song et al., 2000; Nimchinsky et al., 2002; Zucker and Regehr, 2002; Caporale and Dan, 2008; Losonczy et al., 2008; Spruston, 2008; Jia et al., 2010; Mark et al., 2017). SNNs comprise neuromorphic devices in which information transmitted by more than three factors is constrained. Dendrites, as the third factor, are added to pre- and post-synaptic components, and any synaptic state is constrained by the locally surrounding patch of postsynaptic membrane, which contains hundreds of synapses. The pulses with the shortest Δ*t* are identified as getting qualify to play a role in information transmission.

Spike neural networks are closer to the biological brain than 1,2-G networks are. SNNs do not need many units in the network architecture and save energy in contrast to DNNs. However, training SNNs remains a challenge because spike training requires discrete signals without differentiability, and the backpropagation algorithm cannot be directly used, as in DNNs. Recently, deep learning in SNNs has been reviewed in detail (Tavanaei and Maida, 2017), and a hybrid platform has been implemented by integrating DNNs with SNNs (Pei et al., 2019).

### Theoretical weakness: From the energy function to the cost function

For brain-inspired ANNs, what is the dynamic source that drives state changes? Hopfield already answered this question with the energy function of the spin glass model, a solid-state physics model (Hopfield, 1982; Hopfield, 1984). Every unit of the system is a uniform attractor, and each unit’s orientation and position are statistically determined by the attractor’s surface energy and the interaction competition among attractors in the system state space. The energy is the dynamic source of the system and will trend toward global or local energy minimization. The system will reach the stationary state or convergent point when the energy function reaches its minimum. The spin-glass model is too far from the energy metabolism of the biological brain. Brain neurons are polymorphic, and their morphologic appearance and location in the brain are determined by phylogenetic evolution and ontogenetic processes rather than being uniform and stochastically allocated. Brain energy metabolism usually occurs at the basic metabolic level during an organism’s calm state, but it changes to global imbalance and the highest local level with cognitive activity. Therefore, the theoretical attractiveness of the energy function has decayed since the 1990s.

The concept of the cost function or loss function has recently been regarded as the dynamic basis of ANNs (Marblestone et al., 2016; Scholte et al., 2018; Richards and Lillicrap, 2019), but the definition differs among different references, for example, “The idea of cost functions means that neurons in a brain area can somehow change their properties, e.g., the properties of their synapses, so that they get better at doing whatever the cost function defines as their role”; “A cost function maps a set of observable variables to a real value representing the ‘loss’ of the system”; “A cost function is defined as the composition of mathematical operations, e.g., mean squared error”; “A loss function provides a metric for the performance of an agent on some learning task. In a neural circuit, loss functions are functions of synaptic strength”; and “Cost is the partial derivative of the error with respect to the weight.”

All of these definitions and terms are from mathematics or ANN, with very few neuroscientific or psychological implications. How can the cost function be understood by neuroscientists and psychologists? The brain-inspired ANN is an interdisciplinary field, and the main theoretical conceptions should include neuroscientific and psychological implications, at least in terms of brain physiology. Cost, error, weight, partial derivative, mathematical operations, the credit assignment problem, etc.; how many of these come from system neuroscience, integrative neurophysiology or cognitive psychology? As a primitive and basic conception of ANNs is far from the implications of the biological or human brain, it is difficult to establish an interdisciplinary field. Let us review the corresponding conceptions in neuroscience and psychology.

There are different models of energy supply for different brain structure. Large amount of parallel neural fiber in cerebellar did not put on its myelin coat, its energy supply comes only from its cell body; but the neural fiber from cerebrum can get additional energy supply from myelin (neuroglia cell). BOLD signals represent hemoglobin-responsive changes in local brain blood microcircuits or neuroglia assemblies, because hemoglobin cannot directly reach any neuron. Each neuron in the neocortex has 3.72 glial cells, and each Purkinje cell (PC) in the cerebellum has only 0.23 glia cells (Azevedo et al., 2009). In fact, energy supply in brain is, respectively, implemented by area, lamina, column. We suggest that the energy supply in ANNs needs to be improved, so that the energy consumption will be saved.

## Information processing in the brain

Although the conception of information processing did not appear until the 1940s, brain anatomy and neurophysiology had already become important by the end of the 19th century. Through study of the anatomy of the nervous system, scientists had mastered the knowledge of sensory-motor pathways and the visceral autonomic nervous system as well as brain functional localization. The classical neurophysiological theory, in which the brain was regarded as a reflex organ, was founded at the beginning of the 20th century. By the mid-20th century, the brain was regarded as an information-processing organ since electrophysiological techniques provided scientific evidence of neuron firing and postsynaptic potentials (PSPs) in the 1930s–1960s. The brain is now considered an organ in which both neural and genetic information are processed; that is, the brain’s long-term memory is the result of a dialog between synapse and gene (Kandel, 2001). In recent years, transcriptomic expression has been used to classify the cell types of neuron distribution in the neocortex (Kim et al., 2017; Boldog et al., 2018). The concept of neural information processing is understood from four perspectives: the principle of the simultaneous existence of digital encoding and analog encoding, the principle of the simultaneous existence of multiple processing processes and multiple information streams, the principle of circular permutation and coupling between electrical transmission and biochemical transmission in the processing of neural information, and the principle of relevance between neural and genetic information (Shen, 2017). Therefore, neural information processing is more complicated than any communication information or industry information. Of course, the brain-simulated parameters must be simplified to build an ANN model, but the primitive unit, the network architecture, and the operational dynamics should approximate the biological brain.

### Two types of primitive unit vs. an uniform excitatory unit

A series of discoveries in studies of the spinal reflex at the beginning of the 20th century, such as spatial and temporal summation, convergence, divergence, fraction, synaptic retardation, final common path, and reciprocal innervation, accounted for the interaction between excitatory and inhibitory processes (Sherrington, 1906). In particular, the conditioned reflex theory emphasized internal inhibition as a function of the cerebral hemispheres (Pavlov, 1927). Inhibitory neurons were not found until electrophysiological techniques and electro-microscopy were used. A model of the soma and large dendritic fields of a cat’s spinal motor neurons was published, showing that any nerve cell is encrusted with hundreds of thousands of synapses, with a mean diameter of approximately 1.0 μm (Haggar and Barr, 1950). Even when six different synaptic appearances were demonstrated, synaptic features could not credibly be used to discriminate whether a synapse is excitatory or inhibitory (Whittaker and Gray, 1962). The features of inhibitory synapses were judged by inhibitory postsynaptic potentials (IPSPs), and many more feedforward inhibitions, such as presynaptic inhibition and collateral inhibition, were found in brain networks, including the cerebral cortex, cerebellar cortex, and hippocampus (Eccles et al., 1954; Eccles, 1964).

Studies in brain chemical pathways in the 1960s–1970s found several categories of inhibitory neurons on the basis of their released transmitter molecules, such as gamma aminobutyric acid (GABA), glycine, and serotonin, and found that the inhibitory neurons in the neocortex are composed mainly of GABAergic neurons. The neurons in the neocortex can be divided into two categories: interneurons, which make local connections, and projection neurons, which extend axons to distant intra-cortical, sub-cortical and sub-cerebral targets. Projection neurons are excitatory, synthesizing glutamatergic transmitters with a typical pyramidal morphology that transmit information between different regions of the neocortex and to other regions of the brain (Paredes et al., 2016; Cadwell et al., 2019). Molecular neurobiology using transcriptomic techniques investigates the stereotyped distributions of mainly inhibitory cells in the brain and classifies three classes of inhibitory neurons: parvalbumin-positive interneurons (PV^+^ neurons), somatostatin-positive interneurons (SST^+^ neurons) and vasoactive intestinal polypeptide-positive interneurons (VIP^+^ neurons; Kim et al., 2017). Recently, the biological marker for GABAergic neurons in immunocytochemistry and molecular neurobiology has enabled direct classification into three categories: excitatory, inhibitory and non-neuronal cells, such as glial cells. By this method, a group of human interneurons with anatomical features in neocortical layer 1, with large ‘rosehip’-like axonal button and compact arborization (Boldog et al., 2018), was discriminated that had never been described in rodents.

Studies in GABAergic neurons found that the proportion of GABAergic neurons is approximately 15% of the population of all cortical areas in rats, whereas in the primates, the proportion reaches 20% in the visual cortex and 25% in the other cortex. The numbers of inhibitory interneurons have increased during phylogenetic evolution along with the appearance of unique interneuron subtypes in humans (Wonders and Anderson, 2006; Petanjek et al., 2008). In contrast to the prenatal development of excitatory neurons in the human cortex, interneuron production, at least in the frontal lobe, extends through 7 months after birth. GABA concentrations in the occipital cortex of adult subjects, as measured by magnetic resonance spectroscopy, are relatively stable over periods as long as 7 months(Near et al., 2014). In evolutionary history, the cortical projection neurons derive from the dorsal (pallial) telencephalon and migrate radially into the cortical mantle zone; the interneurons generated in the sub-pallium migrate tangentially across areal boundaries of the developing cerebral cortex into the overlying cortex (Mrzljak et al., 1992; Petanjek et al., 2009). The inhibition shapes cortical activity, and inhibitory rather than excitatory connectivity maintains brain functions(Isaacson and Scanziani, 2011; Mongillo et al., 2018; Fossati et al., 2019). There are large numbers of interneuron types with different morphological, electrophysiological, and transcriptomic properties in the human neocortex (Wonders and Anderson, 2006; Petanjek et al., 2008; Kim et al., 2017; Boldog et al., 2018).

Artificial neural networks have oversimplified the inhibitory unit as a supplementary variant of the excitatory process or a dependent variant of the activation function. ANN experts claim that “inhibition need not be relayed through a separate set of inhibitory units” (Kietzmann et al., 2019). Actually, “in the absence of inhibition, any external input, weak or strong, would generate more or less the same one-way pattern, an avalanche of excitation involving the whole population” (Buzsaki, 2006).

ANNs might be comprised of different categories of units with different connecting strength with their target units by different weight shift range 0–1 or 0–(−1). As a result, there are two types of primitive unit: excitatory and inhibitory unit. Each categories of units will be further divided into different types by their threshold activated in different encoding, for example, sensitivity to spike timing: the lower sensitivity(Δ*t* < 10 ms) or the higher sensitivity(Δ*t* < 6 ms). Also the unit types can be classified by pulse frequency to be evoked or by PPR standard.

### Hierarchical small-world architecture with a circular vs. a uniform feedforward network

The structural basis of the brain and spinal reflex is the reflex arc that comprises afferent, efferent and neural centers. The dual neural processes—the excitatory and inhibitory processes—run in two directions in the reflex arc: along a centripetal pathway from the sensory organs, reaching the corresponding brain sensory center, and along a centrifugal pathway returning from the brain to the peripheral effectors. The small-world network comprises a few components, and each component contains a few constituent parts. The biological reflex arc comprises three components, and each component contains 3–5 neurons, for example, a monkey’s food-fetch reflex pathway (Thorpe and Fabre-Thorpe, 2001). The centripetal pathway is an input or afferent component that comprises three neurons from the retina, lateral geniculate body to primary visual cortex (V1), producing visual sense about the stimuli; the bottom-up stream is a perceptual mechanism from V1 to the anterior inferior temporal gyrus, producing visual perception by the ventral visual pathway; the prefrontal cortex to the premotor area of the cortex formats the decision-making mechanism; and the centrifugal pathway is the efferent or output component of the motor cortex (MC) to the spinal cord and finger muscles. In short word, the food-fetch reflex is not only implemented by 4 hierarchical networks with small-world architecture, but also there are many inhibitory modulating mechanisms: a self-feedback loop, the extrapyramidal system, the descending nonspecific reticular system and the cerebellum. The classical specific brain pathways are small-world architectures that have both the attributes of random rewiring and regular local organization; their synaptic path lengths are both the shortest for random networks and the longest for local networks (Watts and Strogatz, 1998; Buzsaki, 2006). Therefore, the efficiency of brain networks is excellent, with both high-speed information processing and saving of network sources.

On the contrary, DNNs are one-way feedforward networks and usually comprise an input layer, hidden layers, and an output layer in which the highest layer does not to be discriminated, and all units are uniform at the initial state or before the training period. The information stream moves in a feedforward or downward manner from the input layer to the output layer. Sometimes, the convolutional algorithm is implemented in all input sets, the hidden layer is comprised more than tens of layers, and whole connections among all units are implemented in layers.

Therefore, we suggest that the network architecture in ANNs need to be improved. As a commenter on an early version of this manuscript noted: “Current ANNs largely ignore anatomical organizations: topographic mapping, precise wiring between brain areas, layers, and cell types. This specific wiring bestows huge computational power with minimal request of energy consumption. For example, the wiring of the olfactory system is very different from that of the visual system. Similarly, the wiring of the sensory system differs drastically from that of the motor system.”

### Biofeedback everywhere vs. only error backpropagation

The nervous system is a typical servo system with many biofeedback mechanisms that exist everywhere in brain networks but are not like error backpropagation in ANNs. Biofeedback can be found in reflex arcs, synapses, dendrites, axons, presynaptic membranes, biochemical transmission, etc. In the centrifugal pathway, recurrent inhibition in spinal networks is usually mediated by another inhibitory neuron, for example, Renshaw cells or their gamma loop between alpha and gamma motor neurons in the spinal reflex arc (Eccles, 1973, 1989). In the sensory-perceptual system, neural information is transformed along the centripetal pathway to the primary area of the sensory cortex, and the bottom-up information stream reaches the highest area of the perceptual cortex. In addition, there are many feedbacks or top-down streams from the highest perceptual or memory areas, and there are concurrent thalamus-cortical connections among the thalamus-cortical layer IV-cortical layer VI-thalamus (Kamigaki, 2019; Egger et al., 2020). This phenomenon is not consistent with the cognitive model rule that a unit can be connected to a unit in a higher layer or to a unit in the same layer but not to a unit in a lower layer (Feldman and Ballard, 1982; Cummins and Cummins, 2000). Recently, some new facts have been reported, such as dual whole-cell recordings from pyramidal cells in layer V of the rat somatosensory cortex, revealing an important mechanism for controlling cortical inhibition and mediating slow recurrent inhibition by SST^+^ neurons (Deng et al., 2020). In sensory experience and perceptual learning, input from the higher-order thalamus increases the activity of VP^+^ neurons and VIP^+^ neurons and decreases the activity of SST^+^ neurons, resulting in the disinhibition of pyramidal cells in the sensory cortex and the LTP effect. Contextual feedback from the higher-order thalamus is helpful in processing first-order sensory information (Williams and Holtmaat, 2019).

In the mechanism of biochemical transmission of neural information, the re-take-up and auto-receptor protein in the presynaptic membrane receives or reabsorbs its released transmitter. Reverse messengers such as NO and CO molecules released by postsynaptic neurons can quickly suppress the transmitter release of presynaptic neurons.

Sum up in a word, we hope that the feedback or recurrent inhibition should be set in everywhere of the whole networks, instead of only error backpropagation.

### The multiple learning mechanisms vs. a uniform weight switch

The fixed reflex arc in the biological brain was established by phylogenetic evolution and provides a basis of unconditioned reflex; temporary connections between neural centers are the basis of the conditioned reflex. How can the temporary connection be established by training or experience? The unconditioned stimulus (US), for example, food, water or a sexual partner, induces a stronger excitatory process in the corresponding brain center; the conditioning stimulus (CS) at first is an unrelated neutral stimulus that usually induces a weaker excitatory process in the corresponding brain center. Temporary connection is the result of the attraction of a stronger center to a weaker center (Pavlov, 1927). Therefore, the CS must be presented first, and the US must follow in an interval of less than a couple of seconds. The conditioned reflex has been called classical conditioning as the physiological basis of unsupervised learning, in which increased connectionist strength takes place mainly among the neurons in the cerebral cortex. For supervised learning, a goal or a standard as the supervisor must be set in advance. The response should be aimed at an exact goal with a quick reaction time or with the refined skill that demands the inhibited actions. The neural connection between the cerebrum and cerebellum is the neural basis of supervised learning. The short−/long-term plasticity of the synapse in the sensory-perceptual neocortex is the basis of perceptual learning.

The causal role of learning is the coincidence in the tempo-spatial space between stimuli, inducing changes in synaptic plasticity. The temporal-difference learning algorithm has been tested, and an essential process in learning and memory is the transformation between spatiotemporal information in the brain (Fitzsimonds and Poo, 1998; Bil and Poo, 2001).

Two kinds of synapses were identified as the cellular mechanism of animal learning in the 1980s, while the biochemical mechanism of learning has been recognized as the molecular configuration of protein: NMDA-receptor protein and adenylate cyclase (Abrams and Kandel, 1988). Two kinds of synapses infer an activity-dependent synapse and a reinforcement synapse between pre- and post-synaptic components. The activity-dependent synapse takes place between two presynaptic components, and the facilitated component then acts on the third postsynaptic neuron, providing the conditioned response (CR). The reinforcement/rewarding synapse brings about the excitatory process in postsynaptic neuron, while the CS and US signals are transferred on the postsynaptic neuron that is facilitated by the two presynaptic components.

Artificial neural networks does not change their methodological or simulating strategy for machine learning, although a part of researchers in ANNs field has accepted the hypothesis on three types of learning (Doya, 2000). There is difficult for them to processing the differences among different types of learning. We suggest to regulate, respectively, the mode of energy supply for different learning network. The key neurons of supervised learning are Purkinje cells in cerebellar, each cell with a rich dendritic tree in a plane, and receive information from a lot of parallel nerve fibers uncovered myelin coat. The nerve fibers do not require additional energy supply from neuroglia cell (myelin coat). The electric source should be a stable lower power for supervised leaning circuit; on the contrary to the ANN circuit for the unsupervised learning. The electric source of the circuit should be a little higher power and can be quickly changed in a range, because pyramidal neuron in neocortex with a long axon and axonal tree in 3-diminssion space. Each pyramidal neuron has about 4 glial cells who work as both a myelin and additional energy supplier during the process of transmitting neural pulses.

### Uniquely executive control mechanism vs. programing action command

So fa, there are 3–4 theories on the executive control mechanism in psychology: the unity and diversity model (Miyake et al., 2000), functional network of prefrontal cortex (Miller and Cohen, 2001) and the distributed executive control (van den Heuvell and Sporns, 2013). The unity and diversity model is also called “three factor model of update, inhibition and shifting or cognitive flexibility,” because the executive control is defined as the cognitive behavior containing updating of working memory, inhibition of unnecessary actions and frequently changing situating demand.

The executive control behavior is required to have mid-grade of inter-correlation among three experimental indices, so that it presents both the independent and common property of the behavior (Miyake et al., 2000). According to updating of working memory, subject is demanded to say the picture name which is demonstrated in the last one of a sequence of 60 pictures, but the sequence will be stopped randomly from time to time. So, the subject has to update his or her working memory without stopping during the process of the sequence running. Inhibition infers to eliminate the interfere for the performance running or inhibits undesired responses. Stroop color words interfere paradigm is usually adopted. A “blue” word written by red color and a “red” word written by blue color are inconsistent interfere items, the subjects are demanded to press a button according semantic blue, inhibiting the response to blue color. Shifting or cognitive flexibility confers that the experimental task will be randomly changed. For example, 2 digits are successively displayed on a screen, then the computational demand is shown either plus or minus.

The experimental models may not completely present human social intelligence. For example, Chinese proverb “show resourcefulness in an emergency” or “turned on by danger” means that working memory can reproduce a creative idea, during dangerous situation.

The small-world architecture may not be available for the social intelligence of the prefrontal cortex, even though it is important for brain network of basically cognitive process. A single cell axon tree of pyramidal neuron has been shown with thick interlaces and even with 60 thousand branches (Buzsaki, 2006; Boldog et al., 2018). The prefrontal cortex is the core structure to collect different information from other brain areas and efficiently processing the complicate information as well as controlling the related behavior. A lot of evidence from monkey and patients with brain injury demonstrates that prefrontal cortex contains different switches to control proactive, retroactive behavior (Hikosaka and Isoda, 2010), many local microcircuits to play a role in cognitive flexibility or shifting, and salience network to regulate interchanging between default mode network and central executive network (Tang et al., 2012; Goulden et al., 2014; Satpute and Lindquist, 2019). The network hubs in the human brain has been reported by the distributed executive control theory(van den Heuvell and Sporns, 2013), the network hubs are different from the other brain structures, there are the compact white matter in macroscopy and finely structured nerve fiber with high density spinous process in microscopy. It is the structure with the qualify to implement integrating information and controlling behavior. To date, the axon trees and their microcircuits as well as long-range output connectionist pathways in the prefrontal cortex has been believed to be the prerequisite of executive control ability. Especially, medial prefrontal cortex (mPFC) contains much more GABA-inhibitory neuron that collect a lot of information from the other neurons, such as Ach neuron in anterior brain, 5-serotonin neuron in thalamus, sensory cortex, and limbic-hippocampus system as well as memory system (Jayachandran et al., 2019; Nakajima et al., 2019; Ren et al., 2019; Sun et al., 2019; Weitz et al., 2019; Onorato et al., 2020). There are uniquely two kinds of neuron in PFC and mPFC: GABA-ergic chandelier cells (ChaCs) and von Economo neurons(VENs). ChCs are the only interneuron subtype that selectively innervates the axon initial segment of pyramidal neurons in frontal cortex (Tai et al., 2019); there are the higher density of VENs in the elderly above 80 year old with higher intelligence (DeFilepe et al., 2002; Evrard, 2018; Gefen et al., 2018).

The programing behavior sequences written by program editor cooperate robots, even though a guiding robot can performances the act of etiquette and answers question from customer. There are not creative idea or contingency approaches. That an auto drive bicycle runs on the street and avoids obstacles presents only human’s sensory-perception-motor ability but not general intelligence.

## Intelligence is a neuro-computational system

Intelligence was regarded as the ability to compute discrete physical symbols when artificial intelligence was founded in 1956; PDP was regarded as neural computation when the second generation of ANNs was propagated in 1986. Here, intelligence might be a compound of multiple computing processes rather than any single computing process. Intelligence is a neuro-computational system, containing the following computations: deterministic computation, stochastic computation, chaotic computation, and the other computation can be implemented by the human brain.

### Deterministic computation

Neuronal excitation is represented by the rate encoding of its discharge, while the postsynaptic potentials implement analog computation by amplitude modulation. The computation involved in combining digital and analog computing composes a deterministic system. The general intelligence is implemented by the classical specific nervous system, mainly through deterministic computation.

#### Population sparse encoding

The human brain comprises 10^11^ cells (approximately 160 billion), and the cerebral cortex comprises 82% of the total brain mass; however, only 19% of all neurons are located in the cerebral cortex, which is mainly responsible for intelligence (Azevedo et al., 2009). Each brain function is implemented only by a part of the neurons; therefore, the human brain implements most intelligent activities by population sparse encoding.

#### Oscillatory encoding

Spontaneous oscillatory encoding is a prerequisite of normal brain function because it provides optimal arousal or vigilance under the mechanism of the nonspecific reticular system.

#### Infra-slow oscillation

The spontaneous fluctuation in brain BOLD signals with infra-slow oscillation <0.1 Hz concerns not only the default mode networks(DMN) but also the cerebellum and salience network (Raichle et al., 2001; Hikosaka and Isoda, 2010; Raichle, 2010, 2015), which remain to be investigated further. ISA is a distinct neurophysiological process that reflects BOLD signals in fMRI, and its spatiotemporal trajectories are state dependent (wake versus anesthesia) and distinct from trajectories in delta (1–4 Hz) activity with the electrical property (Tang et al., 2012; Goulden et al., 2014; Satpute and Lindquist, 2019).

### Stochastic computation

#### Spike time encoding

Even though the principal neurons in the classical specific nervous system have many dendritic branches and an obvious laminar distribution, their axon has only a few buttons, usually fewer than 15–20 (Branco and Staras, 2009; Borst, 2010; Silver, 2010). The neurons seem to meet the spike time encoding by their dendrites as the third factor in synaptic transmission (Borst, 2010; Scholte et al., 2018).

#### Stochastic encoding

Apart from dendritic algorism as the third factor of synaptic transmission, there are more factors that make up pyramidal neurons as a completely stochastic computational component: dendritic tree, axonal tree, laminar distributing effect, interlaced microcircuit interaction, stochastic noise and short-term synaptic plasticity. The multiple factors form hundreds of micrometers of interneuron space to assemble microcircuits (Bil and Poo, 2001; Fossati et al., 2019).

### Chaotic computation

Chaotic computation is the field of “the qualitative study of unstable aperiodic behavior in deterministic dynamical systems.” A chaos system has three essential properties: it must be dramatically sensitive to initial conditions, with highly disordered behavior, and obey some laws that completely describe its motion. There are three computational indices (Faure and Korn, 2001; Tsuda, 2015; Chao et al., 2018; Sathiyadevi et al., 2019): Lyapunov exponent (*λ*), Kolmogorov-Sinai entropy (K), and strange attractors. *λ* is positive if the motion is chaotic; *λ* equals zero if the two trajectories are separate; *λ* is a constant amount if the trajectories are periodic. *K* equals zero if the system is periodic, whereas if it increases without interruption, the system is stochastic, or it increases to a constant value if the system is chaotic. Strange attractors are fractal objects, and their geometry is invariant against changes in scale or size.

### Adaptive computation

Adaptive computation is a category of biological computation formatted in biological evolution. An individual changes him- or her-self to meet context-dependent multitask demands.

#### Adaptive encoding

The prefrontal cortex coordinates cognition, emotion, interpersonal communication, coping with situations, and other complicated mental activities. Adaptive encoding is an algorithmic principle that meets multiple demands for making decisions, planning, monitoring, error finding and revising in a wide range of tasks and successfully implements goal-directed behavior. The encoding feature is expressed mainly in context-dependent computation with chronometric and dynamic adaptation. Its algorithm is multivariate pattern analysis and multi-voxel pattern analysis *via* principal component analysis.

### The emergent computation: A unique basis of human intelligence

Emergent computation is not completely new but has new implications. “Emergent collective computational abilities” appeared in a paper title in Hopfield (1982). In the article, the emergent computation refers to a functional model of creative intelligence in the human brain that has higher energy efficiency or uses less energy to obtain a stronger effect in nervous information processing.

Although dynamic coding (Stokes et al., 2013), abstract quantitative rules (Eiselt and Nieder, 2013), context-dependent computation (Mante et al., 2013), mixed selectivity (Rigotti et al., 2013), content-specific codes (Uluç et al., 2018) reconfiguration of responses (Jackson and Woolgar, 2018), situational processing (Lieberman et al., 2019), and so on have been reported, adaptive encoding cannot cover the crucial property of human social intelligence—creativity. Human beings not only adapt passively to situations but also actively create human society and themselves (Eccles, 1989; MacLean et al., 2014; MacLean, 2016). Therefore, a new mathematical computation that represents actively unique creativity in natural and social environments has existed in the human brain for more than ten thousand years. It might represent the emergent computation.

The efficiency of brain energy cost interchange for the amount of information processing, the partial derivative of the amount of information with respect to energy cost, might be a basic parameter of brain evolution and brain function. A new concept of emergent computing based on the efficiency of brain information processing is suggested as an approach to interdisciplinary theory.

The first principle of emergent computation is the active or initial desire that drives a human to create. The second is higher energy efficiency, meaning trade of more information by a lower energy cost. The third principle implicates the premise of creative intelligence, and the fourth principle encompasses the chronometric effect and spatiotemporal conversion effect. Because energy metabolism inside the human brain is a slow process (second scale), while nervous information changes or external environmental changes are usually rapid processes (millisecond scale), chronometric mechanisms and spatiotemporal conversion are necessary. The fifth principle is recruiting or reusing neurons or physical sources. It not only contains multiple encodings but also may enable more advanced computation that has not yet been identified by the best mathematical authorities. The algorithm of emergent computation might be based on answer of 10 questions as follows.

## Suggestion and the questions to be investigated further

As mentioned above, ANNs, DNNs and CNNs have already made much progresses accompanied by some bugs. Except big energy consumption and larger number of hardware parts, it is very difficult for DNNs to simulate the social intelligence of the human brain, even by increasing the number of hidden layers or designing complicated input sets. The bug might be in the basically theoretical conceptions about the primitive unit, network architecture and dynamic principle as well as the basic attributes of the human brain.

We have given suggestion to ANNs in the article text, the list of suggestion is as follows:

1. the primitive neural node will be substituted by two types of unit: excitatory and inhibitory with different connectionist weight and threshold. For example, sensitivity to spike timing: the lower sensitivity (Δ*t* < 10 ms) or the higher sensitivity (Δ*t* < 6 ms).
2. The feedback or recurrent inhibition should be set in everywhere of the whole networks, ave. instead of only error backpropagation.
3. topographic mapping, precise wiring between brain areas, layers, and cell types. This specific wiring bestows huge computational power with minimal request of energy consumption. Small world network principle should be absorbed into ANN architecture.
4. Energy supply in brain is, respectively, implemented by area, lamina, column. The energy supply in ANNs needs to be improved, so that the energy consumption will be saved. Energy supply modes are different in different learning types, for example between supervised learning with lower energy consumption mainly in cerebellar and unsupervised learning with higher energy consumption in cerebral cortex.
5. the emergent computation as a basis of cognitive science substitutes “PDP-neuro-computation.” PDP is just one of neuro-computation.

Besides ANNs, the studies in brain simulation along biomedical line have already made much progress in recent decades. For example, the Blue Brain project simulated large-scale networks of neocortical micro circuit based on biological brain data, including morphological and electrophysiological properties as well as cell types of bioactive molecule expression (Markram et al., 2015). A reconstructed virtual brain slice was made up. The meso-circuit was 230 mm thick, containing a total of 139,834 neurons. The virtual slice reproduced oscillatory bursts (1 Hz) and displayed a spectrum of activity states. In addition, the project provided a neocortical model with 0.29 ± 0.01 mm3 containing 31,000 neurons, 55 layer-specific morphological neurons. But, the results are unsatisfactory, because there is only 1 Hz bioelectric activity in the simulated brain slice showing its alive state. The problem might be that how do the much neurons organize together? Apart from laminar and column-like architecture, it is necessary to consider the principle of small-world organization (Watt and Strogatz1998) that means a few neurons compose a microcircuit with a definite basically cognitive function, such as sensory-motor circuits. The questions are worth to inquire further into:

1. Except three neuronal encodings: rate, PPR, and spike time encoding described in Figures 2, a specifically dendritic action potentials (dAPs) has been reported (Gidon et al., 2020). The pyramidal neuron in II/III layer of neocortex has an ability of parting off XOR, because it can reproduce dendtric action potentials(dAPs). The dAPs mechanism is worth investigating further. How does such a neuron implement the computational ability to separate XOR function? The task should be finished by a deep neural network. So, it might be new neural encoding principle.
2. Neurogliaform cells (NGFCs) dynamically decouple neuronal synchrony between brain areas (Sakalar et al., 2022), but does neither decrease their own firing nor affect local oscillation. Is its function alike with backpropagation mechanism in BP learning. In the other words, Does NGFCs take a part in implicit information propagation in biological brain?
3. What is the relationship between brain information and brain energy during intelligent activity?
4. What is the relation of energy with computational power? Is there any difference in computational principle between the neocortex and cerebellum?
5. How does neuroglia cell perform in cognitive function and neural energy supply?
6. What is the meaning of mental capacity-limited or affordable processes for conscious cognitive function from the viewpoint of brain energy metabolism?
7. Is there any unique computation in the human brain beyond modern computational mathematics or postmodern mathematics?
8. Is there any other computing principle of the prefrontal cortex beyond adaptive encoding?
9. Is there any other core computing resource of human brain for social intelligence in addition to the axon tree with long-range networks and local microcircuit-to-microcircuit communication in the prefrontal cortex?
10. Is it possible to build a common theoretical basis of cognitive science to promote the interdisciplinary development of brain simulation?

## Author contributions

ZS designed the study. FS performed the literature search. ZS and FS drafted, revised, and wrote the paper. All authors contributed to the article and approved the submitted version.

## Conflict of interest

The authors declare that the research was conducted in the absence of any commercial or financial relationships that could be construed as a potential conflict of interest.

## Publisher’s note

All claims expressed in this article are solely those of the authors and do not necessarily represent those of their affiliated organizations, or those of the publisher, the editors and the reviewers. Any product that may be evaluated in this article, or claim that may be made by its manufacturer, is not guaranteed or endorsed by the publisher.
